# Identification of novel immune checkpoints as targets for cancer immunotherapy

**DOI:** 10.1186/2051-1426-1-S1-P135

**Published:** 2013-11-07

**Authors:** Galit Rotman, Ofer Levy, Amir Toporik, Gady Cojocaru, Liat Dassa, Iris Hecht, Ilan Vaknin, Anat Oren, Zohar Tiran, Nora Tarcic, Sergey Nemzer, Tania Pergam, Amit Novik, Shirley Sameah-Greenwald, Joseph R Podojil, Stephen D Miller, Judith Leitner, Peter Steinberger, Eyal Neria, Zurit Levine

**Affiliations:** 1 Compugen Ltd, Tel Aviv, Israel; 2 Microbiology-Immunology, Northwestern University, Chicago, IL, USA; 3 Inst. Immunology, Medical University of Vienna, Vienna, Austria

## 

Members of the B7/CD28 family of immune checkpoints, such as CTLA-4, PD1 and PDL-1, play critical roles in T cell regulation and have emerged as promising drug targets for cancer immunotherapy. Utilizing Compugen’s predictive discovery platform, we identified novel members of this family that may serve as immune checkpoints. The therapeutic relevance of these proteins was confirmed following the validation of their immunomodulatory properties and their expression in various cancers. Here we present results obtained for two of our novel B7/CD28 family members: CGEN-15001T and CGEN-15022. Fusion proteins, consisting of the extracellular domain of the predicted proteins fused to an IgG Fc domain, display robust inhibition of T cell activation and therapeutic effects in T-cell driven animal models, EAE and CIA. The Fc fused protein of CGEN-15001T also showed enhancement of iTregs induction. Immunohistochemistry studies on a variety of healthy and malignant tissues indicate expression of both molecules in various types of epithelial and hematopoietic cancers, with each protein showing a unique expression pattern. Expression was also detected on tumor infiltrating immune cells. Based on their immunomodulatory activity and expression in malignant and immune cells, CGEN-15001T and CGEN-15022 show potential as targets for cancer immunotherapy.

